# Integrated Disease Investigations and Surveillance planning: a systems approach to strengthening national surveillance and detection of events of public health importance in support of the International Health Regulations

**DOI:** 10.1186/1471-2458-10-S1-S6

**Published:** 2010-12-03

**Authors:** Celine H Taboy, Will Chapman, Adilya Albetkova, Sarah Kennedy, Mark A Rayfield

**Affiliations:** 1 Laboratory Systems Development Branch, Centers for Disease Control and Prevention, Atlanta, GA 30333, USA

## Abstract

The international community continues to define common strategic themes of actions to improve global partnership and international collaborations in order to protect our populations. The International Health Regulations (IHR[2005]) offer one of these strategic themes whereby World Health Organization (WHO) Member States and global partners engaged in biosecurity, biosurveillance and public health can define commonalities and leverage their respective missions and resources to optimize interventions. The U.S. Defense Threat Reduction Agency’s Cooperative Biologica Engagement Program (CBEP) works with partner countries across clinical, veterinary, epidemiological, and laboratory communities to enhance national disease surveillance, detection, diagnostic, and reporting capabilities. CBEP, like many other capacity building programs, has wrestled with ways to improve partner country buy-in and ownership and to develop sustainable solutions that impact integrated disease surveillance outcomes. Designing successful implementation strategies represents a complex and challenging exercise and requires robust and transparent collaboration at the country level. To address this challenge, the Laboratory Systems Development Branch of the U.S. Centers for Disease Control and Prevention (CDC) and CBEP have partnered to create a set of tools that brings together key leadership of the surveillance system into a deliberate system design process. This process takes into account strengths and limitations of the existing system, how the components inter-connect and relate to one another, and how they can be systematically refined within the local context. The planning tools encourage cross-disciplinary thinking, critical evaluation and analysis of existing capabilities, and discussions across organizational and departmental lines toward a shared course of action and purpose. The underlying concepts and methodology of these tools are presented here.

## Introduction

The International Health Regulations (IHR[2005]) [1] requirements parallel a number of biosurveillance programs’ core elements and represent a language that is acceptable to leadership around the world. They focus on establishing processes and building national capacity for reporting of any event that could be perceived as a threat to global health security. Additional standards and/or guidance provided by the International Organization for Animal Health (OIE) and The United Nations’ Food and Agriculture Organization (FAO) represent complementary frameworks to engage country leadership on the animal health and food security fronts. Furthermore, the tripartite strategic alignment published by FAO-OIE-WHO in 2010 [2] and the One Health Initiative [3] offer additional directives to improve coordination at the animal, human and ecosystems interfaces and reiterate the commitment to coordinate global activities to address health risks. These global mandates compel the strengthening of partner countries’ detection and response systems in a holistic and systematic manner.

Enhancing disease surveillance systems requires the integration of multiple technical disciplines and stakeholders in a structured and informed design process. The final design and set of interventions ought to differ based on the context and challenges existing locally, therefore requiring customizable and adaptable implementation strategies to ensure the feasibility and effectiveness of the interventions. Because surveillance and preparedness require coordination and collaboration among various programs, first line providers (veterinarians or clinicians), epidemiologists, information system specialists and laboratory personnel, design efforts must consider each of these groups’ needs, capabilities, limitations, logistical assets, budgetary realities and legal requirements. The Integrated Disease Investigations and Surveillance (IDIS) tools enable planning efforts for a robust and functional capability backbone on which specialized tactical net works aimed at preparedness and rapid response can be built. The success of this approach lies in execution of a system-wide design process that fosters communication and collaboration amongst the multiple stakeholders operating within a surveillance system, two elements that are pivotal for building effective and agile coordinated national response to local and international public health emergencies.

## Methodology

As mentioned, the IDIS tools are set up to guide a design effort aimed at developing a comprehensive and sustainable local solution for improved disease detection and surveillance. The format compels informed discussions across human and animal networks, bridging together the clinical and veterinary worlds with epidemiological, laboratory and program elements. The plans provide a framework and tools for multidisciplinary teams of experts to apply a broader “system thinking” approach [4,5] when working to improve existing biosurveillance systems. Their primary purpose is to provide a deliberate planning process that will capture system-wide critical information that can be later used for immediate and long-terms goals development (e.g. modifications of testing strategy and reporting, training, strategic planning, operational research, procurement/infrastructure and regulatory framework, and targeted interventions).

The tool contains two parts: (1) a template and guidance for the system-wide review of the existing bio-surveillance environment and (2) a series of pathogen-specifi c plans, organized in syndrome clusters to reflect the importance of having differential diagnostic capabilities in order to rule-in or rule-out specific diseases. The rule-in or rule-out testing strategies will be dependent on the country’s existing capability, endemicity of the pathogen, infrastructure requirements, biosafety regulations and the benefit to risk ratio of adding capacity at various tiers of the disease surveillance networks.

Emphasis is placed on determining cross-cutting weaknesses or obstacles such as overall stewardship and management issues, lack of established standards of operations, or critical resources issues (human or material), as these tend to often be underestimated and can lead otherwise well-planned interventions to failure [6]. In addition, it is clear that even the simplest change within the system may have a butterfly effect as health systems are dynamic, complex, and interrelated systems with the capacity to amplify small changes. Improved mechanisms for communication, enhanced understanding of the system interfaces and processes, and ultimately a system that can effectively go through iterative processes and organizational improvements are the true measures of success.

### System assessment

Because surveillance and biosecurity covers a wide range of technical disciplines, a diverse group of qualified international experts working with the program implementers may be called upon to support discussions during the early phase of engagement. The resulting operational assessments provide the baseline on which to delineate the system design. Gathering information related to the regulatory framework (i.e., the current standards, statues, and regulations that control the surveillance system); the organizational components of the health system; the surveillance and epidemiology capacity and the framework for outbreak and emergency preparedness; workforce competency and human resources capacity at each tier of the health system; key research activities and global partnership engagement in the country or region prior to the planning process is essential.

CBEP and CDC use a collection of tools to evaluate and gather the information on existing capabilities, namely modified versions of the “National Inventory of Core Capabilities for Pandemic Influenza Preparedness and Response” [7], the “OIE tool for the Evaluation of Performance of Veterinary Services” [8] and the IHR Monitoring Framework [9] as they provide monitoring and evaluation frameworks to assess improvements of the core capacities over time.

The information gathered should reach a level of granularity that is consistent with one’s ability to make informed recommendations during the design process. It should capture existing strategies or initiatives being implemented in country by the diverse health sectors, and outline potential conflicts that could arise from the implementation of system-wide modifications.

Early information-gathering visits are critical and may be done in parallel with workshops focused on bringing managers responsible for the various sectors of the health system together to discuss existing connectivity and systems approach to improving biosurveillance and health outcomes. Forums should be established that bring together various stakeholders to work toward a comprehensive understanding of the existing landscape and reflect on where the system could and should be improved, and how to best coordinate and leverage partner ship in country. Recognized outside experts from these various disciplines may act as moderators of these discussions to shape the dialogue.

### Mapping of existing detection and surveillance systems

At the national level, surveillance and detection systems for human and animal infectious diseases are typically under the responsibility of different departments and ministries. Understanding the inter-relationship between existing networks of these ministries and their interaction with the private sector is critical to mapping multi-sectorial linkages and coordination. These findings will inform the needs for broader involvement in discussions about system strengthening and sentinel detection, reinforcing the importance of linking syndromic recognition, case definitions, laboratory capacity, testing algorithms, and reporting mechanisms. This knowledge should be an early product of system evaluation activities.

Mapping of the system linkages can be done in writing or using flow diagrams. The key is to capture enough details to make the information relevant and usable during the planning process. Examples are provided within the tools to guide this activity.

### System recommendations and design

Focused discussions with senior leadership need to outline the pros and cons of making changes to the existing system, address financing and governance implications, and identify sustainability strategies. These discussions may require performing additional fact finding and may be facilitated by the use of case scenarios of routine infectious disease reporting and testing, and of a rapid response to a suspected case or cluster of cases. A high level of detail is required at this stage to avoid creating parallel systems, unfeasible or impractical requirements, or unrealistic expectations. The outcome of this phase should be an agreed upon architecture of inter-connected networks capable of monitoring, detecting, assessing, and reporting events, while being sustained within the constraints defined during the evaluation process.

The recommendations should go beyond detection capability, and require addressing the impact and value added of implementing changes. Larger system-wide issues should be addressed early on in order to improve the overall effectiveness of focused interventions. Strengthening management, use of recognized standards and best practices that will guide the entire network, as well as focus on workforce development and strategic workforce planning are critical elements of the establishment of sustainable systems. While planning for the design of a sustainable inter-related detection system, it is important to consider the high costs of responding to false alarms, while balancing the risks of delaying a case investigation. Finding a balance between faster, often less-sensitive but affordable diagnostic tools, and more specific and more complex methods is critical, as these decisions have a tremendous impact on the effectiveness of the system.

The IDIS planning tools provide design guides to lead the team(s) through an iterative evaluation and decision making process. Each iteration of the process will address a lower level of detail within the design until the capacity building objectives and implementation strategies are clearly defined for their program. In the early stages of the process, high level relationships between ministries and major facilities involved in the detection and reporting process are articulated. During follow-up iterations, the operations and standards that govern the system may be addressed, until facility level operational plans and standard operating procedures and policies are developed.

The pathogen-specific templates in IDIS provide a framework to move from one element to the next to point out the inter-relationship between disciplines and support a consensus building environment where clinical or veterinary program, epidemiology and laboratory leader ship can understand the importance of the continuum of activities to improve surveillance outcomes. Templates have been developed for a number of pathogens of interest to the biosecurity community, and are organized to summarize the existing and desired capacities as follows: 1) the existing capability section contains the agent summary, country-adapted case definition, local epidemiological landscape, current system mapping and testing strategies, standard operating procedures (SOPs), and reporting structures; 2) the desired capability section provides background information on tests available for the detection of the pathogen, and a section that summarizes the recommended changes discussed during the planning process. These pathogen-specific templates are designed to prompt the user to develop and link a complete set of activities and documents that are customized to the specific local requirements.

The laboratory plays a critical role in ruling in or out the etiological cause of an event. Even though there are more and more rapid field tests available, their use remains limited to selected groups of infectious agents. Determining the need for referral of samples and the role and responsibility of the country’s laboratory network will vary from setting to setting and from pathogen to pathogen. The ability of the local system to absorb and maintain a specific technology or laboratory diagnostic test is governed by such critical variables as cost, logistics, human resources, and clinical/veterinary outcomes. Laboratory capability remains essential for confirmation, characterization and further preventive actions, but may not need to be available in country [10]. Linkages to existing reference centers may be a better alternative in some settings and should not be ignored, nor should political and regional sensitivities. The continuing issues associated with the reporting of a false positive result may be a motivation for laboratory confirmation capability to be established in a country, but care needs to be given to the ability of the local system to produce reliable and timely data so that alerts can be trusted and acted upon effectively.

### Products and outcomes of IDIS planning

The key products and outputs from these interactions and the planning process include agreements on:

• *Syndromic clusters for early assessment of events:* Starting with syndromic characteristics is helpful as it allows categorizing diseases by clusters, reinforces the notion that differential diagnosis needs to be in place in order to recognize an event in a timely fashion, and reinforces the need for continuous clinical, veterinary and basic laboratory competencies of the first line providers.

• *Case Definitions:* The use of standard case definitions increases the specificity of reporting and improves the comparability of events reported from different geographical areas. [11] Establishment of case definitions is therefore critical to the surveillance and detection systems, more so in the case of events of global importance. The case definitions represent a body of work in itself, and if not already existing, will require the attention of subject matter experts working with country leadership to develop locally-adapted case definitions on which the system can be built. The presence of laboratory and program leaders during the development of the case definition is often helpful as it engages each group to better understand their role and responsibilities as well as provide critical information to guide the recommendations.

• *Testing strategy:* The testing strategy is the summary of the sequence of tests to be conducted at each tiered level of laboratory (local, regional, national) based on existing and anticipated capabilities. It represents the rule-in and rule-out laboratory strategies that are decided upon in order to confirm a case. For this reason, the capacities and competencies for each service level should be defined in advance of the implementation of the surveillance network. Facilitating discussion of the limitations of each of the testing options by epidemiologists and laboratory managers as a team is critical. The intrinsic properties of a diagnostic test or a technology will influence the usefulness of the data, and are often neglected topics of discussions that epidemiology and laboratory communities need to concertedly address in advance.

• *Testing algorithms:* The algorithms implemented are derived from testing strategies based on the procurement system and availability of resources. A testing strategy may lead to multiple testing algorithms depending on locally available test kits and reagents, and available resources.

• *Standard Operating Procedures (SOPs):* Decisions regarding testing algorithms are directly linked to procurement needs and standard operating procedures. SOP templates have been developed for a majority of the techniques used in the detection of especially dangerous pathogens of interests to CBEP. Although the development of locally-owned and adapted SOPs represent the lowest level of detail in the design process, institutionally-based review and adaptation processes for the establishment of these core documents are critical to standardize procedures within an institution or network. These documents provide facility specific guidance in the execution of specific tests and procedures, but retain critical processes that allow for comparability of the data for appropriate decision making. They are key elements in the establishment of a quality management system aimed at increasing reliability and accuracy of the data generated by the network.

These agreed-upon elements provide the building blocks for the integrated implementation of a quality management system reaching passed the institution-level to the surveillance system as a whole. Monitoring and evaluation strategies and metrics have been developed by the program to assess improvement of the performance and outcomes of the system as a whole, and are based on internationally recognized tools [7-9].

### Indirect outcomes

In addition to the tangible design outputs, the overall planning process will inform the need for modifications to the foundation of the system and build within the partner country new mechanisms to facilitate continuous improvement. These outcomes typically fall under one of the following three categories:

• *Workforce capacity and competency*: Personnel with skills and ability to recognize unusual disease patterns and surveillance systems linkages among first line providers, epidemiologists and laboratory experts.

• *Organizational and systems capacity*: Essential infrastructure, resources, and strategic planning, as well as inter-relationships, management, organizational structure, laws, policies, rules and regulations that govern the system.

• *Information and data system*: Organized reporting and flow of information.

These indirect outcomes and processes can be leveraged by the partner to address other disease detection pro grams needs due to their cross-cutting nature. Mechanisms and pathways for broader reforms can be under taken. Communication strategies can focus on improving vertical and horizontal linkages throughout the system, with the impacts of change in one sector being under stood and accounted for by the supporting sectors. A strategic framework for the country can then be developed around which international technical assistance can be harmonized.

## Conclusion

CBEP and CDC have partnered to develop methodologies and tools to guide the design and execution of locally-owned capacity building activities to improve disease surveillance systems. These tools focus on a systems approach to integrated disease investigations and surveillance planning. The complexity of multiple networks and vertical programs within a country requires a holistic approach to ensuring reliability and timeliness across the entire health system. Often, linkages between each level of the health system and the processes that should ensure recognition and communication of a suspicious event are non-functioning or fall within a vertical network unable to react or appreciate the importance of an event.

The international community has long bemoaned the difficulties of the vertical approaches on which they often rely. The IHR(2005) [1] and the FAO-OIE-WHO collaboration [2] are refreshing strategic directions that create a paradigm shift and demand a holistic commitment to health strengthening by the global community. By emphasizing sound governance and leadership, quality systems, management competencies targeted at comprehensive and complex networks, workforce development and retention and adoption of internationally-recognized standards, the international community supports countries in developing a more robust and adaptable backbone on which specialized programs and disease specific networks can be successfully established.

The lack of adequate mechanisms for linking programs and leveraging shared interests has often resulted in CBEP and CDC working at cross purposes in the past, competing for the attention and talents of the same individuals in partner country, while providing uncoordinated directives. Such narrowly focused efforts may also have failed to appreciate potential ripple effects they have created across the larger system of networks, or recognize the limitations of larger networks to absorb the changes being implemented. This resulted in a waste of time and efforts, the inability of countries to leverage or sustain the improvements, and/or missed opportunities to positively affect health outcomes.

By investing up front in thoughtful holistic design efforts that bring a broader stakeholder community to the table, the system becomes more aware of its own multifaceted nature and complexity, and those who strive to assist partner countries in continuously improving the outcomes of the surveillance activities can do so synergistically. The systems approach presented here, if done correctly, will strengthen the interfaces within and between networks, building core competencies and capabilities within the system, and focus on the fundamental cross-cutting elements governing the networks. Focusing technical assistance on supporting the development of comprehensive, nationwide, systematic and robust core elements and processes can ensure specif c disease detection challenges are addressed more effectively and can rely on a well-articulated and functioning architecture; an architecture that can learn and grow to respond adaptively to a dynamic environment in which new and unanticipated events can be detected and assessed—a capability at the heart of the IHR.

## Abbreviations

CBEP: Cooperative Biological Engagement Program; CDC: Centers for Disease Control and Prevention; FAO: Food and Agriculture Organization; IDIS: Integrated Disease Investigations and Surveillance; IHR: International Health Regulations; OIE: International Organization for Animal Health; SOPs: Standard operating procedures; WHO: World Health Organization.

## Competing interests

The authors declare that they have no competing interests.

## Authors’ contributions

CHT: concept of IDIS; development and revisions of the IDIS tools; first draft, review and approval of the manuscript. LWC: concept of IDIS; review of the IDIS tools, first draft, revisions and approval of the manuscript. AA: development and revisions of the IDIS tools, review and approval of the manuscript. SK: concept of IDIS and review and approval of the manuscript. MAR: concept of IDIS, development and revisions of the IDIS tools, review and approval of the manuscript.
